# Hydatidiform mole resulting from sexual violence

**DOI:** 10.1186/1755-7682-5-8

**Published:** 2012-02-21

**Authors:** Jefferson Drezett, Flavia Cella Kurobe, Cecília Tomiko Nobumoto, Daniela Pedroso, Marcia Blake, Vitor E Valenti, Luiz Carlos M Vanderlei, Fernando Adami, Franciele M Vanderlei, Sandra Dircinha Teixeira de Araujo Moraes, Maria Auxiliadora F Vertamatti, Alberto OA Reis, Carlos Bandeira de Mello Monteiro, Renata C Rossi, Luiz Carlos de Abreu

**Affiliations:** 1 Laboratório de Escrita Científica, Departamento de Morfologia e Fisiologia, Faculdade de Medicina do ABC, Av. Príncipe de Gales, 821, 09060-650 Santo André, SP, Brazil; 2 Departamento de Fonoaudiologia, Faculdade de Filosofia e Ciências, Universidade Estadual Paulista, UNESP, Av. Hygino Muzzi Filho, 737, 17525-900 Marília, SP, Brazil; 3 Departamento de Fisioterapia, Faculdade de Ciências e Tecnologia, Universidade Estadual Paulista, UNESP, Rua Roberto Simonsen, 305, 19060-900 Presidente Prudente, SP, Brazil; 4 Rua Pedroso Alvarenga 1255 conjunto 64, CEP 045331 012 Itaim Bibi, SP, Brazil

## Abstract

**Background:**

Hydatidiform mole (HM) is characterized by abnormal proliferation of human trophoblast with producers functioning tissues of human chorionic gonadotropin. It can evolve with ovarian cysts tecaluteínicos, hypertension of pregnancy or hyperthyroidism. The incidence of HM is variable and its etiology poorly known, associated with nutritional factors, environmental, age, parity, history of HM, oral contraceptives, smoking, consanguinity or defects in germ cells. There is no reference in literature on HM resulting from sexual violence, objective of this report.

**Method:**

Description of two cases of HM among 1146 patients with pregnancy resulting from sexual violence treated at Hospital Pérola Byington, São Paulo, from July 1994 to August 2011.

**Results:**

The cases affected young, white, unmarried, low educated and low parity women. Sexual violence was perpetrated by known offenders unrelated to the victims, under death threat. Ultrasound and CT of the pelvis showed bulky uterus compatible with HM without myometrial invasion. One case was associated with theca lutein cysts. The two cases were diagnosed in the second trimester of pregnancy and evolved with hyperthyroidism. There was no hypertension, disease recurrence, metastasis or sexually transmitted infection.

**Conclusion:**

The incidence of HM was 1:573 pregnancies resulting from rape, within the range estimated for Latin American countries. Trophoblastic material can be preserved to identify the violence perpetrator, considering only the paternal HM chromosomes. History of sexual violence should be investigated in cases of HM in the first half of adolescence and women in a vulnerable condition.

## Background

Gestational impairments are important factors to be investigated [1,2], since it affects children development [3-5]. The hydatidiform mole (HM) is a variant of gestational trophoblastic disease, a generic term that brings together different conditions of proliferation of trophoblastic epithelium, with abnormal functioning tissue producing human chorionic gonadotropin (hCG) [6]. The gestational trophoblastic disease includes forms of HM, invasive mole, gestacional choriocarcinoma and placental site trophoblastic tumor, according to anatomoclinical criteria [7].

The choriocarcinoma is the most severe form of the disease with potential for invasion and metastasis, affecting every 20,000 pregnancies [8]. The etiology of HM is unknown. Studies associate it with nutritional deficiencies, environmental conditions, herbicides, parity, smoking, extremes of maternal age, intrauterine device, inbreeding, viral infections, oral hormonal contraception, history of HM or germ cell defects [9]. The incidence of HM varies. In Latin American countries may have frequency as low as 1:3.846 pregnancies, while in Asian countries its incidence reaches 1:99 [10,11]. This divergence probably results from a heterogeneous way of collecting data on population or hospital basis.

Sexual violence has a high prevalence and commits primarily young women of reproductive age [12-15]. Although the risk of pregnancy in these cases does not exceed 5% in each exposure, it is estimated that about 30.000 forced pregnancies are carried out only in the U.S. every year [16]. In addition, even facing the large number of cases of pregnancy resulting from sexual violence, there is no reference in literature on its association with HM. A literature review indicating the occurrence of HM in age that features sex crime, according to the legislation of each country, would justify the communication to the competent authority and the investigation of sexual violence. Therefore, this study was undertaken to describe two cases of HM after rape.

## Method

Report of two cases of HM from 1146 women and adolescents with pregnancy resulting from sexual violence treated at Pérola Byington hospital, São Paulo, from July 1994 to August 2011, by consulting the medical records. All procedures were approved by the Ethical Committee in Research of our Institution (Protocol number: 003/08). The literature review to verify the association between sexual violence and HM consulted database Journal Citation Reports (JCR-ISI), Medical Literature Analysis and Retrieval System Online (Medline), Scientific Electronic Library Online (SciELO) and Latin American and Caribbean Literature in Health Sciences (LACLHS). The search strategy used DeCS/MeSH descriptors with syntax ("Hydatidiform Mole" [MeSH]) AND "Rape" [MeSH] and the syntax ("Hydatidiform Mole" [MeSH]) AND "Child Abuse, Sexual" [MeSH Terms] [17,18].

## Case report

**Figure 1:**
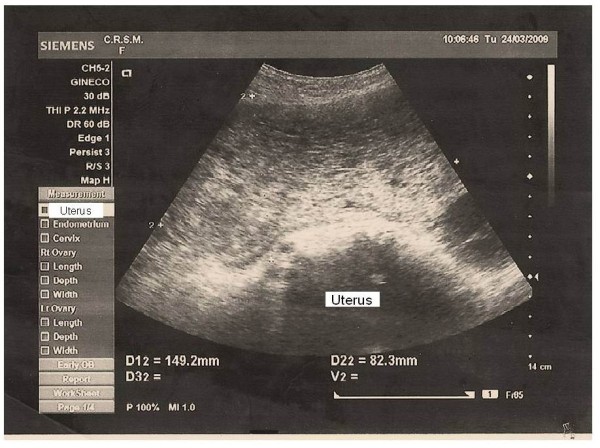
**Typical sonographic appearance of hydatidiform mole (case 2)**.

**Figure 2:**
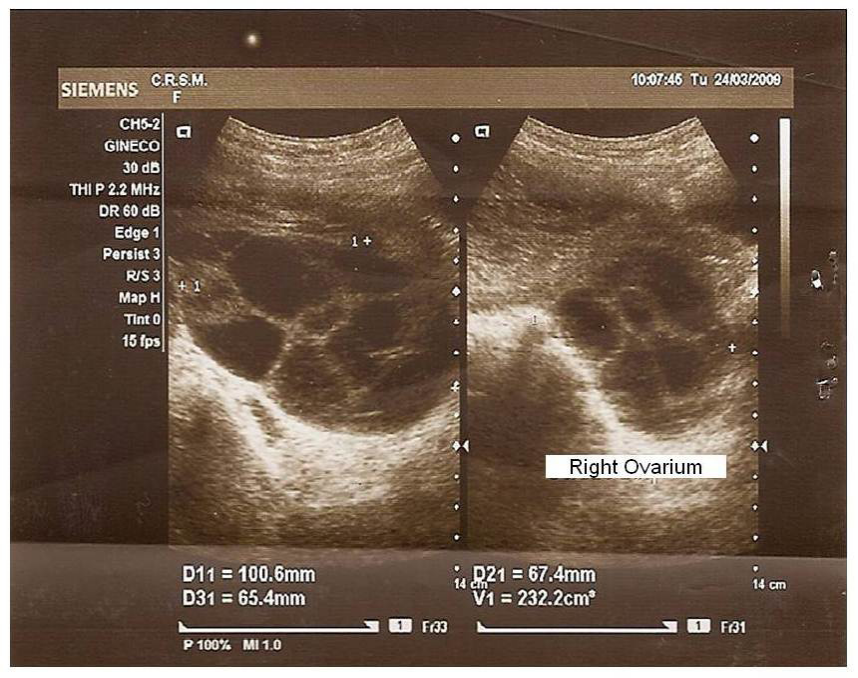
**Ultrasound aspect of the right ovary with 232 cc, showing large theca lutein cysts (case 2)**.

No items were identified in databases relating HM and sexual violence. The incidence of HM was 1:573 pregnancies resulting from rape. The assaults were reported by two young, white and unmarried. Sexual violence was committed by a known abuser unrelated to the victim by threat of death. Ultrasound and non-contrast-enhanced CT of the pelvis showed bulky uterus and compatible with HM content, with no signs of invasion of the myometrium or adjacent structures (Figure 1). In one case, the HM was associated with theca lutein cysts with right ovary with 232 cc and left with 227 cc (Figure 2). The cysts showed complete spontaneous remission four months after uterine evacuation.

In Table 1 we observe the synthesis of two cases of hydatidiform mole resulting from rape.

**Table 1:** Synthesis of two cases of hydatidiform mole resulting from rape

	Case 1	Case 2
**Age**	22 years	13 years
**Parity**	Two	Zero
**Gestational age**	13 weeks	16 weeks
**Uterus volume**	740 cc	920 cc
**Initial Beta hCG (mIU/ml)**	320.080	960.320
**Theca lutein cysts**	No	Yes
**Serum TSH levels**	0,011 mcg/dl (0,4-5,5)	0,013 mcg/dl (0,4-5,5)
**Evolution with HDP**	No	No
**Tomography of Thorax**	Normal	Normal
**Uterine evacuation**	Manual vacuum aspiration	Manual vacuum aspiration
**Anatomopathological**	Complete hydatidiform mole	Partial hydatidiform mole

cc (cubic centimeters) TSH (thyroid-stimulating hormone)

HDP (Specific Hypertension Disease in pregnancy)

The cases evolved with hyperthyroidism and the spontaneous normalization of thyroid-stimulating hormone (TSH) occurred in the first month after treatment. There was no hypertensive disorders of pregnancy (HDP). There were not identified significant changes in blood count, coagulogram, oxaloacetic transaminase (SGOT), pyruvic transaminase (SGPT), urea, creatinine, glucose, calcium, and thyroid ultrasound. Clinical and laboratory parameters, including CT control of the chest and pelvis, and the dose of beta hCG fraction, showed that cases were not associated with recurrent or metastatic disease. None of the cases underwent prophylactic chemotherapy.

Serology for hepatitis B and C, HIV, syphilis and HTLV I and II were negative when enrolled at the service and for the six-month period for monitoring of sexual violence. There were no positive sexually transmitted infections in cervical scrapes and vaginal content. There were no complications that could compromise the reproductive future of the patients. The cases were formally reported to the police by the patients and the rape against the adolescent was reported by the hospital to the governmental agency for protection.

## Discussion

In this study, we described the evolution of the two cases reported of HM after rape. The physical attacks were reported by two young girls. Also, sexual violence was committed by a known abuser.

Based on our data, the both cases presented hyperthyroidism and there was no HDP. Moreover, the spontaneous normalization of TSH was observed in the first month after treatment. We reported changes in blood components, such coagulogram, SGOT, SGPT, urea, creatinine, glucose, calcium, and thyroid ultrasound. According to the literature, the HM can be complete when there is no formation of embryonic or fetal tissue, characterized by diffuse hyperplasia of the cytotrophoblast and syncytiotrophoblast. Partial or incomplete fetal material can be identified along tissue with stromal trophoblastic inclusions [19,20]. HM usually leads to vaginal bleeding in the first quarter and disproportionate uterine growth in relation to gestational age, accompanied by nausea and frequent vomiting, or hyperemesis gravidarum.

Based on clinical and laboratory parameters, including CT control of the chest and pelvis, and the dose of beta hCG fraction, the cases were not associated with recurrent or metastatic disease. None of the cases underwent prophylactic chemotherapy. In 15% to 25% of cases, the high concentration of hCG induces the formation of bulky ovarian theca lutein cysts, with more than 60 mm in diameter [21]. Early HDP is observed in 27% of complete HM and may end in eclampsia [22]. In addition, the HM can progress to full malignant forms of gestational trophoblastic disease in 20% of cases. Hyperthyroidism is found in less than 10% of the cases of HM, resulting from cross-stimulation of the TSH receptors by alpha chain of hCG [23].

Our findings are in agreement with the literature, since women at the extremes of reproductive age are more likely to develop HM. The relative risk is 7 to 10 times higher among those with more than 40 years [24]. However, the absolute number of HM cases after 40 years is small, due to decreased fertility and reduced frequency of pregnancy in this age group. The risk is 2 times higher in the first half of adolescence, although there are few records of HM under 15 [25].

The aging of the eggs may explain in part the increased susceptibility to abnormal gametogenesis and fertilization in women over 40 years, but does not justify the HM in younger women. Most studies suggest that the higher maternal age increased the incidence of choriocarcinoma [26]. In addition to maternal age as a recognized risk factor, personal history of HM increases up to 40 times the risk of the disease in following pregnancies [24]. Parity does not seem to have a decisive influence on the incidence, with 12% to 78% of cases of HM between nulliparous. Few studies suggest that the incidence grows with parity, or that relates to increased risk of choriocarcinoma [26].

Information on other risk factors are limited and conflicting, and may vary when the HM is complete or partial. However, in Asian countries there is sharp decrease in the incidence of HM in recent decades related to the improvement of socioeconomic conditions of the population, suggesting the importance of environmental or nutritional factors in its etiology [27].

In our study, the both cases were formally noticed to the police by the patients and the rape against the adolescent was reported by the hospital to the governmental agency for protection. Sexual violence is not associated directly or indirectly, with most known risk factors for HM. There is exception for age and parity, since the majority of rape victims are young women or adolescents, often nulliparous [12]. There is also no evidence that male factors have influence on the etiology of HM except inbreeding. Therefore, it cannot be attributed to the sexual violence's author who is unrelated to the victim a risk factor for HM, which can be corroborated by the incidence of HM 1:573 pregnancies resulting from sexual abuse observed in this report, within the limits of variation in Latin American countries [24,26].

However, the relationship between the perpetrator and HM may have important legal implications. In 90% of complete HM the karyotype is 46XX, and 10%, 46XY. In partial HM triploid karyotype is shown in 90% of cases, 69XXX or 69XXY with tetraploidy in 10%. All of these are exclusively paternal chromosomes [19,20,26]. The proper preservation of part of the molar tissue allows to keep for a long period, genetic material that can be used for DNA analysis and determination of paternity. This issue is relevant to the identification and accountability of perpetrators of sexual violence, often not guilty by the courts for lack of evidence [12].

The absence of reports in the literature linking HM to sexual violence must be interpreted with reservation. HM cases reported in the first half of adolescence could be due to sexual violence, to the extent that the laws of many countries typifies sexual activity as a crime under certain age limits [25]. In Brazil, for example, it is considered a crime to have sex with children under 14 years. The underreporting of sexual violence, estimated at almost 90% of cases, can also interfere with the lack of records. The embarrassment and the threat of death can lead many women to choosing not to disclose to health professionals that the HM arises from sexual violence [12].

## Conclusion

In summary, the occurrence of HM in age that features sex crime, according to the legislation of each country, justifies the communication to the competent authority and the investigation of sexual violence. Our findings indicate that reported cases of sexual violence in HM require additional health professionals care with an investigation of sexually transmitted diseases, psychological treatment and support for legal action seeking and protection.

## Competing interests

The authors declare that they have no competing interests.

## Authors' contributions

All authors participated in the acquisition of data and revision of the manuscript. All authors determined the design, interpreted the data and drafted the manuscript. All authors read and gave final approval for the version submitted for publication.
